# Comment on: “Which nostril should be used for nasotracheal intubation with Airtraq NT®: the right or left?”

**DOI:** 10.3906/sag-1903-31

**Published:** 2020-06-23

**Authors:** Raghuraman M SETHURAMAN

**Affiliations:** 1 Department of Anesthesiology, Sri Venkateshwaraa Medical College Hospital & Research Centre, Ariyur, Puducherry India

**Dear Editor,**

I read an article published in the February 2019 issue, which compared the ease of nasal intubation using Airtraq through the right or left nostril [1]. I greatly appreciate the efforts of the authors for conducting this thought-provoking study. I wish to provide my reflections on some points which do not correspond to the viewpoints of the authors of this study [1].

The concept of comparing the right versus left nostril for the ease of nasal intubation based on the literature [2] quoted by authors is understandable. However, the point of contention is that we cannot conclude that choosing the right nostril would make the intubation safer and quicker in all patients, because Takasugi et al. had observed in their retrospective study that left nostril had to be chosen in 15% of the cases following failure through right nostril due to anatomical variations [2]. Indeed, it is difficult to conclude which nostril is more patent clinically, as the clinical predictors do not correlate well with the endoscopic findings [3]. Hence, it is better to prepare both nostrils as anesthesiologists usually do and try the one which is more patent, not as per the surgeon’s preference (oral/nasal can be of surgeon’s preference, not right/left nostril) as mentioned in the introduction section of the current study [1]. In case of difficulty, the other nostril can be tried. 

The authors had incorrectly mentioned in the results section that the insertion times were “comparable” instead of “significantly differing” between groups as the P-value was <0.001. 

The Sellick maneuver and the external laryngeal manipulation (ELM) is not the same as mentioned in the “Results” section. They are entirely different as the former one is applied over the cricoid cartilage for preventing the regurgitation, whereas the latter one (ELM) is applied to improve the visualization of vocal cords by manipulation on the thyroid cartilage.

In the discussion section, the statement that studies and case reports are available that had successfully performed all nasal intubations through the right nostril with Airtraq NT totally mismatches with regard to one of the three references cited for it. The study by Xue FS et al (cited as Ref # 7 in the current study [1]) was an observational study that had only analyzed the feasibility of Airtraq NT for orotracheal intubations (not for nasal intubations as mentioned in the article) and found that it was successful in all the 42 patients [4]. Another statement that the Airtraq NT would be produced with a left channel in the coming days to make it easier for nasal intubations through the left nostril also does not match with the three references cited for it.
